# Species Discrimination, Population Structure and Linkage Disequilibrium in *Eucalyptus camaldulensis* and *Eucalyptus tereticornis* Using SSR Markers

**DOI:** 10.1371/journal.pone.0028252

**Published:** 2011-12-07

**Authors:** Shanmugapriya Arumugasundaram, Modhumita Ghosh, Sivakumar Veerasamy, Yasodha Ramasamy

**Affiliations:** 1 Division of Plant Biotechnology, Institute of Forest Genetics and Tree Breeding, Coimbatore, Tamilnadu, India; 2 Division of Genetics and Tree Breeding, Institute of Forest Genetics and Tree Breeding, Coimbatore, Tamilnadu, India; University of Umeå, Sweden

## Abstract

*Eucalyptus camaldulensis* and *E. tereticornis* are closely related species commonly cultivated for pulp wood in many tropical countries including India. Understanding the genetic structure and linkage disequilibrium (LD) existing in these species is essential for the improvement of industrially important traits. Our goal was to evaluate the use of simple sequence repeat (SSR) loci for species discrimination, population structure and LD analysis in these species. Investigations were carried out with the most common alleles in 93 accessions belonging to these two species using 62 SSR markers through cross amplification. The polymorphic information content (PIC) ranged from 0.44 to 0.93 and 0.36 to 0.93 in *E. camaldulensis* and *E. tereticornis* respectively. A clear delineation between the two species was evident based on the analysis of population structure and species-specific alleles. Significant genotypic LD was found in *E. camaldulensis*, wherein out of 135 significant pairs, 17 pairs showed r^2^≥0.1. Similarly, in *E. tereticornis*, out of 136 significant pairs, 18 pairs showed r^2^≥0.1. The extent of LD decayed rapidly showing the significance of association analyses in eucalypts with higher resolution markers. The availability of whole genome sequence for *E. grandis* and the synteny and co-linearity in the genome of eucalypts, will allow genome-wide genotyping using microsatellites or single nucleotide polymorphims.

## Introduction

Linkage disequilibrium (LD) estimation is currently in focus for plant breeding because of its application in association mapping of quantitative and adaptive traits in natural populations. Association mapping or association analysis is a method to study the relationship between phenotypic variation and genetic polymorphism [1] with high potential to establish marker–trait associations based on the LD present across the genome under study. In forest trees, although bi-parental mapping population based quantitative trait loci (QTL) identification has been practiced widely, association mapping holds promise as a strategy to implement marker assisted selection of quantitative traits for efficient tree breeding. It is advantageous for plants with long gestation period due to the assay of broader allelic variation in a single study [2]. Association mapping is influenced by the characteristics such as genetic diversity, population structure and the extent of linkage disequilibrium existing in the selected panel [3], [4]. The extent of LD varies among the populations within the species and also across the genome of the species under study [5], [6]. The pattern and extent of LD determines the number of DNA markers required for successful identification of markers linked to a particular phenotypic variation. In polygenic traits, the phenotype is governed by multiple genes and identifying the candidate gene becomes the prerequisite for LD mapping and such information is lacking for many of the economically important species. Other than marker assisted selection for quantitative traits in undomesticated forest trees, the extent of LD and its distribution pattern has the potential to enhance and accelerate genetic resource management activities, including gene conservation [7]. Much of the research on the extent and distribution of linkage disequilibrium has been reported in humans, animals and annual crop species [8]–[10]. However, in forest tree species, LD estimation was reported in conifers like pines [11], douglas fir [12] and in hardwoods like *Eucalyptus* [13] and *Populus* [4], [14], [15]. Both, neutral DNA markers such as simple sequence repeats (SSRs) and candidate gene based single nucleotide polymorphisms (SNPs) were utilized to understand the parameters of LD. Except for few, most of the LD studies in forest trees used SNPs in candidate genes. In *Eucalyptus* hybrids, LD was estimated with random amplified polymorphic DNA (RAPD) markers [16] while SSR markers were used for LD estimation and the significant allelic associations were recommended for early selection of individuals for mass propagation or clonal testing in *Pinus radiata* [17]. In *Vitis vinifera* an out-crossing perennial species with high diversity, the utility of SSR markers were demonstrated for genome wide analysis [18], [19].

Eucalypts are one of the predominant tree species exploited for the paper pulp production. The tropical eucalypt plantations in countries like India are mainly occupied by *Eucalyptus camaldulensis* and *E. tereticornis* due to their wider adaptability to various types of edaphic and climatic conditions. In natural locations, these species occur in sympatry, particularly in Queensland region (Australia) and overlapping flowering period facilitates interspecific hybridizations [20]. Possibilities for interspecific hybrid generation in these species for utilizing hybrid vigour are enormous. Genetic diversity analyses of these eucalypt species with neutral markers like amplified fragment length polymorphisms (AFLPs) and inter simple sequence repeats (ISSRs) revealed higher levels of genetic variability within populations than among the populations [21]–[23]. Microsatellite based genetic diversity analysis along with geographic trends of distribution could differentiate 7 subspecies in *E. camaldulensis* [24]. As in many other forest tree species, QTL identification in eucalypts, essentially depends on interspecific hybrid generation, pseudotestcross strategy based linkage map construction and localization of QTLs on the consensus map [25], [26]. Studies conducted in *Eucalyptus* species showed that LD decays within several hundreds of base pairs and suggesting the possibilities of much finer mapping resolution in LD based association mapping [13]. In this study, we provide the first investigation on the LD estimation and decay of the *Eucalyptus* using multiallelic SSR markers. Our specific objectives were to (1) discriminate the species and investigate population structure within selected germplasm of *E. camaldulensis* and *E. tereticornis* (2) determine the extent and genomic distribution of LD between pairs of SSR alleles to analyze the upcoming opportunities for association mapping in eucalypts.

## Results

### Microsatellite Allele diversity

Hundred and nine microsatellite loci from various eucalypt species (*E. grandis, E. nitens* and *E. tereticornis*) were cross amplified in *E. camaldulensis* and *E. tereticornis* and 62 primers (56.9%) amplified one or two bands and others showed either no amplification or spurious pattern. A total of 1067 alleles were detected in 93 individuals amplified with 62 microsatellites. The summarized data on polymorphic information content (PIC), major allele frequency, observed and expected heterozygosity for the accessions of *E. camaldulensis*, *E. tereticornis* and its hybrids are given in Table 1. The PIC value denotes the relative informativeness of each marker and in the present study it ranged from 0.24 to 0.93 with an average of 0.86 (Table 1). Both *E. camaldulensis* and *E. tereticornis* showed high mean PIC value of 0.84. To explore genetic diversity among genotypes within the species, estimates of observed and expected heterozygosity and average number of alleles was also calculated. The observed heterozygosity varied from 0.44 (Eg24, LG 3) to 0.95 (Embra35, LG 1), with an average of 0.87. Similarly, the expected heterozygosity values ranged from 0.36 to 0.92 with the average of 0.69 for all eucalypt accessions. The detailed data for each microsatellite loci is given in supporting information (Table S1). The number of alleles for each locus ranged from 5 to 30 with average of 17.2 for all 93 accessions. The highest number of alleles, 30 and 27 were found at locus Embra35 and Embra207 respectively. Twenty SSR loci amplified between 20 and 30 alleles while three loci amplified fewer than ten alleles. The lowest number of five alleles was amplified by Eg24 (data not shown). Within *E. camaldulensis*, the number of alleles per locus ranged from 4 to 24 with an average of 14.2. In *E. tereticornis* the number of alleles ranged from 5 to 24 with an average of 14.7. F1 hybrids of *E. tereticornis* showed very low number of alleles when compared to the pure species but had higher variability in heterozygosity estimate, indicating the recent recombination of two different genomes (Table 1).

**Table 1:** Range of the number of alleles per SSR, polymorphism information content, gene diversity and heterozygosity for *Eucalyptus camaldulensis* (EC) and *E. tereticornis* (ET) accessions (with their standard errors, SE).

Parameters	EC	SE	ET	SE	F1 Hybrids	SE
Number of	4–24	14.2±4.5	5–24	14.7±4.1	2–10	6.5±1.99
alleles						
Major Allele	0.10–0.68	0.23±0.12	0.10–0.78	0.25±0.13	0.20–0.85	0.41±0.18
Frequency						
PIC value	0.44–0.93	0.84±0.1	0.36–0.93	0.84±0.11	0.24–0.86	0.68±0.16
Gene Diversity	0.49–0.93	0.86±0.09	0.38–0.93	0.85±0.10	0.26–0.87	0.71±0.15
Heterozygosity	0.37–0.95	0.69±0.16	0.37–0.95	0.69±0.16	0.00–1.00	0.67±0.16

### Species discrimination and Population structure

Species-specific SSR alleles were identified from 40 *E. camaldulensis* individuals, 35 *E. tereticornis* individuals and 8 landraces (Indian selections). Among the 62 SSR loci analysed 55 loci (89%) were polymorphic and 7 loci were monomorphic across all the three groups and 17 SSR loci were monomorphic between the two species. Analysis of 38 microsatellite loci (61%) for the presence of most common alleles with GDA software showed that 23, 14 and 38 SSR alleles were specific for *E. tereticornis, E. camaldulensis* and the landraces respectively (Table 2). The most common alleles of 13 SSR loci in the landraces were present in either of the two species revealing that they could be putative hybrids of *E. camaldulensis* and *E. tereticornis*.

**Table 2:** Most common species-specific SSR alleles of *E. camaldulensis*, *E. tereticornis* and landraces.

Locus name	Linkage Group	*E. camaldulensis*	*E. tereticornis*	Landraces
Embra11	1	138	136	**138**
Embra56	1	160	148	148*
Embra6	1	140	148	**140**
Embra70	1	158	162	154
Embra12	1	134	134/142	**134**
Embra35	1	232/254	240	262/230
Embra100	1	238	250	246
En10	1	144	140	150
Embra172	2	296	294	292
Embra43	2	102	114	**102**
Embra207	2	236	228	220
Embra227	3	312	292	318
Embra122	3	136	144	124
Embra77	3	318	308	286/**318**
Embra19	4	150	186	138/148/168/176
Embra66	4	148	166	170
Embra36	4	254	248	256
Embra179	4	136	132	130
Embra41	5	194	208	198
Embra54	5	138	128	**138**
Embra9	5	128	132	130
Embra24	5	152	148	148*
Embra5	5	130	126	124
Embra8	6	148	146	158
Embra50	6	126	122	**126**
Embra25	6	270	258	274
Embra20	6	150	132	152
Embra226	7	190	178	188
Embra119	8	138	136	**138**
Embra17	9	226	242	242*
Embra204	9	142/162	148	156
Embra58	9	160	142	158
Embra10	10	136	134	138
Embra23	10	126	120	**126**
Embra29	11	260	284	**260**
Embra39	11	134	138	130

Landraces alleles in bold indicates the sharing with *E. tereticornis*, while with asterisk symbol indicates the sharing with *E. camaldulensis*, while the others are specific for landraces only.

**Figure 1:**
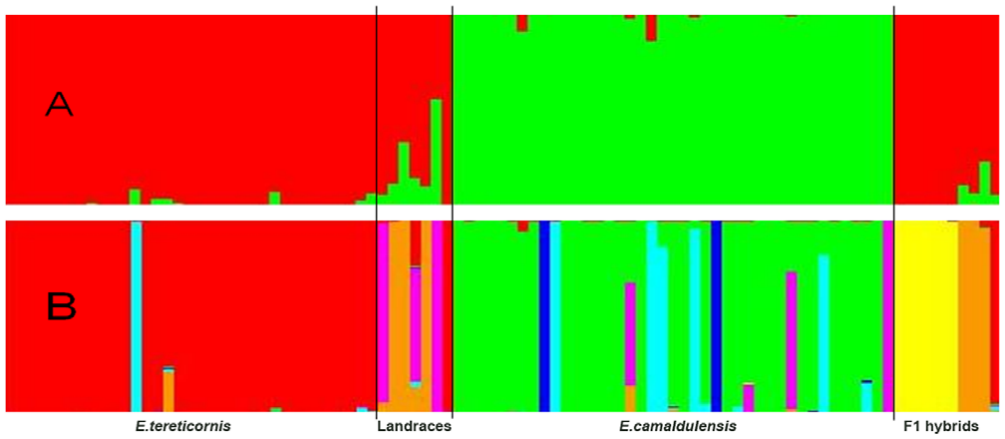
Population structure of *Eucalyptus* accessions used in the study obtained with STRUCTURE software. Each individual is represented by a thin vertical color. Each color represents an accession, and the color of individual represents their proportional membership in the different populations. A and B depicts the population clustering for K = 2 and K = 7 respectively.

The genetic structure of the eucalypts collection was analyzed with the STRUCTURE program with 62 SSRs. Initially the two species were considered as individual populations and hence STRUCTURE was used not to determine the numbers of populations but to assign individual genotypes to the two populations. In K = 2, *E. tereticornis* (including hybrids and landraces) formed a group and other accessions of *E. camaldulensis* formed a separate group (Fig. 1A). We have conducted additional exploratory analysis with higher K and the Evanno's K supported K = 7. However in K = 7, *E. tereticornis* showed subdivision of the population and *E. camaldulensis* did not separate into subpopulations (Fig. 1B). Analysis of molecular variance revealed that the *F_ST_* values between the two populations was 0.019 (*P* = 0.001) and partitioning of the variation was highest within individuals (74%). Similar amount of variance was detected with K = 7 estimated from STRUCTURE, but with higher *F_ST_* values between the populations (*F_ST_* = 0.04; *P*<0.000) (Table 3) and very low differentiation between the individuals of hybrid population (*F_IS_* = −0.07463; *P* = 0.782). However, estimation of LD was conducted by considering two species as individual group of populations.

**Table 3:** Partitioning of genetic variation in *Eucalyptus* accessions.

Source of variation	Degrees of	Sum of squares	Percentage of
	freedom (df)		variation
Among populations	4	273.79	-
Among individuals	87	2849.5	4.03
Within populations	92	1868.5	22.5
Within individuals	183	4991.8	73.46

### Linkage disequilibrium analysis

The eucalypts species were considered separately for estimation of LD, since speciation increases linkage disequilibrium. Assuming the absence of Hardy-Weinberg equilibrium (HWE) in the selected population, the unphased genotypic data was used to estimate genotypic LD or composite LD coefficient (Δ_AB_) between most common pairs of alleles among all loci using GDA software. In *E. camaldulensis* out of 135 significant pairs (df = 1, *P*<0.05), 18 pairs showed r^2^ in the range of 0.05 to 0.09 and 17 pairs showed r^2^≥0.1. Similarly, in *E. tereticornis* out of 136 significant pairs, 18 pairs showed r^2^≥0.05 to 0.09 and 18 pairs showed r^2^≥0.1. In the *E. camaldulensis* accessions, pairwise r^2^ estimates among 62 loci varied from 0.00 to 0.133 with a mean of 0.09 and in *E. tereticornis* r^2^ varied from 0.00 to 0.62 with a mean of 0.012.

Haplotypic interallelic LD among all loci distributed over 11 linkage groups for the two species were estimated using reconstructed haplotypic data as implemented in MIDAS software by avoiding the assumption of HWE. The most frequent alleles represented by N/Y and Y/Y combinations (4248 allelic pairs for *E. tereticornis* and 4098 allelic pairs for *E. camaldulensis*) were considered for LD estimation. In *E. tereticornis* and *E. camaldulensis* accessions 46 and 15 allelic pairs were significant respectively (Yates corrected χ^2^; df = 1, *P*<0.05) and showed interallelic r^2^>0.1. No allele pair with r^2^<0.1 was significant. The minimum and maximum interallelic r^2^ value for *E. camaldulensis* was 0.11 and 0.51 respectively with the mean of 0.19. Similarly, in *E. tereticornis*, the minimum and maximum interallelic r^2^ was 0.16 and 0.41 with the mean of 0.25.

**Figure 2:**
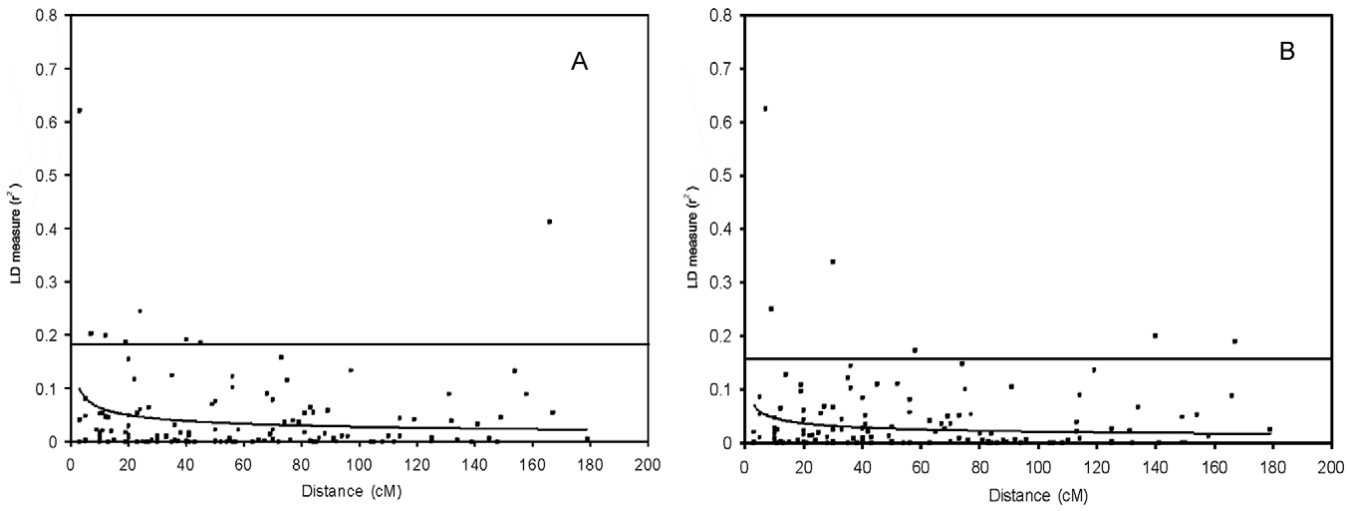
LD decay plot of SSR allele pairs as a function of genetic distance (in cM) for eucalypts. The curves illustrate LD decay based on the nonlinear regression of r^2^ on genetic distance and the horizontal lines indicate the baseline r^2^ values based on the 95^th^ percentile of the distribution of r^2^ values. A, B – Genotypic LD measured with GDA for *E. camaldulensis* and *E. tereticornis*.

The LD decay plots of the genotypic data are displayed in Fig. 2. The 95^th^ percentile of the distribution of these estimates was used as a population-specific threshold for this parameter as an evidence of linkage. In the present study, the population specific threshold value of genotypic LD (r^2^) was = 0.17 and 0.14, and haplotypic r^2^ was = 0.39 and 0.31 for *E. camaldulensis* and *E. tereticornis* respectively. The r^2^ values above these threshold were probably caused by genetic linkage [1]. However, a NLR curve fitted on the r^2^ estimates for genotypic data (Fig. 2A and B) was below the 95^th^ percentile baseline as well as r^2^ = 0.1 indicating the rapid decay of LD in eucalypts. The haplotypic LD measured as interallelic LD with MIDAS showed r^2^>0.1 (data not shown). To determine the relevance of these alleles (r^2^>0.1) for association analysis, identification of more number of SSR alleles with higher LD may be required. Between the species, accessions from *E. tereticornis* showed more number of allele pairs in LD than *E. camaldulensis*.

## Discussion

### Molecular diversity in eucalypts

The transferability of SSR loci among the species of eucalypts is amply represented [27], [28] and it was highly successful in the present study for *E. camaldulensis* and *E. tereticornis*. Analysis and characterization of microsatellites in *Eucalyptus* species have detected loci with highly variable number of alleles, heterozygosity levels and PIC values [29]. Allelic variability observed in these two species was very similar to other species of eucalypts. Brondani et al. [30] reported allele number of 14.34 and heterozygosity 0.87 in the species belonging to subgenus *Symphyomyrtus*. In *E. camaldulensis*, the PIC and heterozygosity of SSR loci ranged from 0.48 to 0.99 (average of 0.84) and 0.25 to 0.90, respectively [28]. However, in *E. camaldulensis*, Butcher et al. [24] reported 40 alleles for the locus Embra11 with allele size range varying from 72–172 bp and concurrently in the present study 22 alleles in the range of 124–150 bp were identified.

### Population structure and species discrimination

In eucalypts, the predominance of natural hybrids is well documented and landraces are considered as putative hybrids [20], [31]. Further, few of the provenances of *E. tereticornis* were reclassified under *E. camaldulensis* and many provenances show mixed morphology between the two species [20]. It could be due to the natural hybridization occurring at the seed source locations in Australia and unclear delineation of the distribution boundaries between the species [20], [25]. In this study, SSR analysis of *E. camaldulensis* and *E. tereticornis* with 38 loci could differentiate both the species due to the presence of species-specific most common alleles. Although limited number of landrace accessions were used, the presence of SSR alleles belonging to both the species was obvious, suggesting that these SSR markers could be used to identify pure species and their hybrids.

### Linkage disequilibrium

To the best of our knowledge, this is the first study in eucalypt species on the LD estimation using SSR markers. Different measures of LD are available for biallelic markers, however use of such measures with SSR markers tend to decrease the LD by averaging the allelic effect as a single estimate [32]. In heterozygous species, the interpretation of LD becomes complicated because of the non availability of haplotypic phases of the alleles [32]. In this study, interallelic LD was estimated using composite linkage disequilibrium (GDA) [33] and gametic linkage disequilibrium (MIDAS) [34]. Based on the NLR curve it is clear that the LD in eucalypts decays faster as in other tree species such as *Populus nigra* [35]. Low levels of LD in the genome may require an exponentially increasing population size for detection of marker-trait associations [36]. The low LD detected in this study is expected because compared to crop species, perennial out-crossing tree species has a higher effective recombination rate, which leads to a rapid decay of LD [12]. Rapid LD decay was reported in many other out-crossing tree species such as *Pseudotsuga menziesii* var. *menziesii*, where the SNP marker based LD was very low (r^2^<0.05) [37]. In *Populus nigra* decay of r^2^ with distance in *CAD4* gene was observed at about 16 bp [35], while the previous studies reported the decay between 50–500 bp [4], [38]. In *Eucalyptus globulus* 20 wood quality candidate genes was analysed using SNP markers and LD was estimated to decay rapidly except in few genes where LD extended beyond 500 bp [39]. Recently, it was observed that *Populus nigra* and *P. balsamifera* genome had rapid LD decay across the gene sequences [11], [15]. In some of the out-crossing domesticated crops such as ryegrass [10] and maize [36] also the mean r^2^ was well below 0.05 when estimated with SSR markers. In perennial fruit species like *Prunus persica* and *Vitis vinifera*, having a history of domestication and breeding showed a long range LD among the cultivated varieties [40], [18] while in wild grapevine, *Vitis vinifera* L. subsp. *silvestris* LD decayed rapidly, with r^2^ values decreasing to 0.1 within 2.7/1.4 cM for genotypic/haplotypic data [19]. In the present study, pairwise genotypic LD measured by r^2^ was very low (mean r^2^ = 0.039 and 0.038 for *E. camaldulensis* and *E. tereticornis* respectively) (Fig. 2A and B), since the 62 SSRs had an average marker interval of approximately 24.8 cM according to the integrated consensus map of Brondani et al. [25]. Further, the higher number of allele pairs in LD observed in *E. tereticornis* accessions could be due to the existence of structure in the analyzed population.

The haplotype based interallelic LD, employed in human and animal populations for LD and association analysis [41]–[43] was calculated in the two species of eucalypts. Although limited number of allele pairs showed significant r^2^, the interallelic LD estimated in *E. camaldulensis* and *E. tereticornis* was high (mean r^2^ = 0.19 and 0.22), indicating the possibilities of using SSRs for association analysis . Similarly, Inghelandt et al. [36] observed that multiallelic SSRs have four to five times higher mutation rate than SNPs suggesting higher power of SSRs to detect LD than biallelic SNPs, if marker density is ignored. Further, Stich et al. [44] showed a clear advantage of SSRs over AFLPs to detect LD in a population with short history of recombination. Verhaegen et al. [16] estimated LD of eucalypt hybrids using RAPD markers and reported significant relationship between the cumulative number of marker alleles in the parents with the full-sib family performance for various traits.

Most of the LD estimation studies in tree species are conducted on candidate genes [12], [14] and LD estimates in non-genic regions are still unavailable [38]. Further, the development of locus-specific assays for single nucleotide polymorphisms (SNPs) is difficult unless the specific gene sequence is isolated from the particular species. In maize, an outcrossing crop, genome-wide sample of 47 SSRs demonstrated higher levels of LD than SNPs in candidate genes [45]. In humans, LD detected using microsatellite markers were significantly wider, about 3 Mb apart than those detected using SNPs with only about 0.5 Mb [46]. In *Pinus radiata*, LD was tested in full sib families created with limited number of pollen and seed parents (45 individuals), using 34 SSR markers and significant correlation was observed between trait and marker loci [17].

### Opportunities for association mapping

The basis for linkage disequilibrium based association analysis in plant breeding is to ultimately develop markers tightly linked to trait loci or to identify exact causal loci for marker assisted selection [47]. This can be achieved by whole genome scans using a large number of neutral markers either associated or not associated with a phenotypic trait or by selecting particular genes as candidates for testing more specific associations with putatively correlated phenotypic traits. On using neutral markers like SSRs for LD estimation it was reported that high ratio of LD between unlinked and adjacent loci for SSR markers is a major disturbing force in gene mapping, which indicates association between markers and genes located on different chromosomes resulting in a high rate of false positive detections of marker trait association [36]. Further, SSRs could be more powerful for association mapping if they were available in the genome with the same density as SNP markers. However, the SSR markers employed in the present study would not be adequate for association analysis, because of insufficient marker density for the germplasm evaluated. Owing to the difficulties associated with conventional QTL mapping, LD present in the extant population of interest is exploited and hence it is highly attractive for tree species. Several SSR markers have been linked with QTLs for important traits in various eucalypt species using different species combinations. Nevertheless, conserved QTLs have been located on homeologous linkage groups of the taxonomically related species [48], [24] and several candidate genes co-located to QTL positions controlling different traits [49]. Low LD in eucalypts promises a higher resolution in genome-wide association mapping, however, many more markers are required to span the whole genome. Given the moderate genome size (∼650 Mb) and the availability of whole genome sequence of eucalypt species, it should be possible to develop high density SSR markers for characterizing the genome. The information generated in the past research on QTL mapping could be used in eucalypts by understanding the pattern and extent of LD in the QTL hot spots. Genome wide association mapping in combination with eQTL data and whole genome marker data will yield significant insight into the genetic architecture of complex traits and help to elucidate the contribution of gene expression to natural trait variation [50].

In conclusion, the presence of species-specific alleles and population structure of the sample suggests that clear species delineation occur and hence separate management of these two species is highly essential in introduced countries to maintain species purity and progeny heterosis. The microsatellite information generated in this study has broadened our understanding on the linkage disequilibrium of the two important species, *E. camaldulensis* and *E. tereticornis*, which has high implications in genetic improvement. It provides an understanding of how LD varies in the genome of outcrossing forest trees, hitherto available only with small genomic regions.

## Materials and Methods

### Plant material

The background of the accessions belonging to *E. camaldulensis* and *E. tereticornis* used in this study is available as supporting information (Table S2). The germplasm for analysis was selected from one to few individual per provenance because the goal was to evaluate the overall diversity in the selected germplasm which was assembled to form the association panel for adventitious rooting traits. The first group consisting of *E. camaldulensis* included 40 accessions from Australia. The second group consisting of *E. tereticornis* had 53 accessions with 35 sourced from provenances of two countries (Papua New Guinea and Australia), 8 landraces of India and 10 F1 putative interspecific hybrid trees, where the maternal parent is *E. tereticornis* (henceforth referred as F1 hybrids).

### Microsatellite amplification

Total genomic DNA was extracted from the juvenile leaves of the selected trees according to Balasaravanan et al. [22]. Eighty eight SSR markers located on the eleven linkage groups selected from the SSR set developed and mapped by Brondani et al. [26] from *E. grandis*, ten primers from *E. grandis* and *E. nitens* mapped by Thamarus et al. [51], 10 EST- SSR primers designed by Yasodha et al. [52] from *E. tereticornis* and 1 SSR (EMCRC 47) from *Corymbia citriodora* subsp. *variegata* mapped by Shepherd et al. [53] were screened for PCR amplification in all *Eucalyptus* accessions. The PCR amplification was carried out in 10 µl volume containing 0.4 µM of each primer, 1 unit of *Taq* DNA polymerase, and 0.4 mM of each dNTPs, 1× buffer (with 200 ng BSA) and 10–20 ng of template DNA. The PCR amplification was carried out for 5 min at 94°C, 30 cycles of 1 min at 94°C, 60 or 30 sec at the primer specific annealing temperature, 2 min at 72°C, and 7 min at 72°C for final extension. Annealing temperatures varied from 48°C to 60°C, to amplify specific microsatellite markers. PCR products were size-separated using an 5% denaturing polyacrylamide gels of size 21 cm×50 cm (Sequi-Gen GT System, BIO-RAD, USA) containing 7 M urea and 1× TBE buffer, and visualized by silver staining. The PCR products of the 93 accessions were run randomly for each primer and plate to avoid scoring errors among plates. Sixty two primers producing one or two clear bands were selected for further analysis (Table S1).

### Species discrimination and Population structure analysis

Presence of most common alleles in 40 individuals of *E. camaldulensis*, 35 individuals of *E. tereticornis* and the 8 landraces were identified by GDA software to differentiate both the species and its landraces. The microsatellite data were generated in this study were scored (bp) manually using the 50 bp size standard (MBI, Fermentas, USA). Observed and expected heterozygosity, allele frequencies and polymorphic information content (PIC) values for each primer were calculated using the PowerMarker software [54]. Assigning of the individuals belonging to two species was inferred using the model based clustering algorithm implemented in STRUCTURE v. 2.1 [55] employing prior population with no admixture model under correlated allele frequencies. K values between 2 and 10 were evaluated using 50000 burnin period and 50000 MCMC replications. Each K value was run 10 times. The distribution of Ln P(D) did not show a clear mode for the true K on the graph and hence to select the best K value Evanno's [56] correction was calculated. Analysis of molecular variance and *F_ST_* estimation was conducted using the program Arlequin, v. 3.0 [57]. Although STRUCTURE results showed 7 groups, five populations were only considered for AMOVA analysis because 3 STRUCTURE identified groups had each only 2–4 individual and hence considered as single population. Therefore the degree of freedom among populations was 4 (Table 3).

### Linkage disequilibrium estimation

The phases between alleles at two heterozygous SSR loci were unknown, hence composite linkage disequilibrium (LD) coefficients (Δ_AB_) between pairs of common alleles at two loci were calculated. Calculations were based on Weir's method [58] implemented in GDA 1.0 software [33]. These calculations were normalized to obtain the interallelic Weir's correlation coefficient [59] r^2^_AB_, as in Barnaud et al. [18]. The LD estimation does not assume HWE and collapses alleles into most common alleles. Fisher's exact test (3200 permutations) implemented in the version was used to estimate the significance (*P*<0.05) of disequilibrium between all pairs of loci.

The interallelic LD computation was performed using the software MIDAS (Multiallelic Interallelic Disequilibrium Analysis Software) [34] which considers the multiallelic SSR markers in calculating r^2^ between all possible interallelic associations. EM algorithm is used to estimate haplotype frequencies from phase unknown genotype data. The software stratifies the sample of two locus haplotypes into N/N, N/Y and Y/Y indicating rare alleles (N) and most frequent alleles (Y). In this study N/Y and Y/Y were only considered for LD estimation as the rare alleles may skew the linkage disequilibrium estimates [34]. A chi-square test with Yates correction was used to identify the significant p-value.

The decay of LD over genetic distance was investigated by plotting pairwise r^2^ values against the distance (cM) between markers on the chromosome. Map positions of all mapped SSRs were based on the consensus map developed by Brondani et al. [25] for *E. grandis* X *E. urophylla* and Thamarus et al. [51] for *E. globulus*. The consensus map was selected in this study because a high degree of co-linearity and synteny between the genetic maps of various eucalypts species was demonstrated including *E. tereticornis* [48]. To describe the relationship between LD decay and genetic distance among all the loci, the parametric 95^th^ percentile of that distribution was taken as a population specific critical value of r^2^, beyond which LD was likely to be caused by genetic linkage. The overall decay of LD (r^2^) with physical distance (cM) among the SSR loci was evaluated by nonlinear regression (NLR) [36]. Curves of LD decay with genetic map distance for SSR loci using genotypic data were fitted by NLR with Prism 4.0 software (GraphPad Prism Program, GraphPad, San Diego, CA, USA). The model of Hill and Weir [59] was used for NLR to fit the expectation of r^2^ with our data.
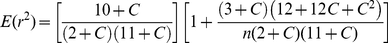
where C is the population recombination parameter (C = 4Nc; N being the effective population size and c the recombination fraction between the loci pair considered) and C was replaced with C X genetic distance in cM.

## Supporting Information

Table S1 **SSR loci used in this study showing information on linkage group, annealing temperature, amplification range, major allele frequency, polymorphic information content, observed and expected heterozygosity.**
(DOC)

Table S2 **Details on eucalypts accessions used in this study.**
(DOC)
