# Supplementary material for: Species Discrimination, Population Structure and Linkage Disequilibrium in Eucalyptus camaldulensis and Eucalyptus tereticornis Using SSR Markers
Source: PLoS One. 2011 Dec 7;6(12):e28252. doi: 10.1371/journal.pone.0028252 (PMC3233572; doi:10.1371/journal.pone.0028252)
Supplement: Table S1 — SSR loci used in this study showing information on linkage group, annealing temperature, amplification range, major allele frequency, polymorphic information content, observed and expected heterozygosity. (DOC) [file pone.0028252.s001.doc]

**Table S1: SSR loci used in this study showing information on linkage group, annealing temperature, amplification range, major allele frequency, polymorphic information content, observed and expected heterozygosity.**

| **SSR loci** | **LG** | **Annealing** | **Amplification range (bp)** | | **Major allele** | | **Polymorphic Information** | | **Heterozygosity** | | **Gene Diversity** | |
| --- | --- | --- | --- | --- | --- | --- | --- | --- | --- | --- | --- | --- |
|  |  | **temperature (°C)** |  | | **frequency** | | **Content** | |  | |  | |
|  |  |  | **EC** | **ET** | **EC** | **ET** | **EC** | **ET** | **EC** | **ET** | **EC** | **ET** |
| Embra11 | 1 | 56 | 108-158 | 108-158 | 0.12 | 0.24 | 0.92 | 0.89 | 0.66 | 0.81 | 0.92 | 0.89 |
| Embra56 | 1 | 56.5 | 120-174 | 120-174 | 0.20 | 0.27 | 0.86 | 0.87 | 0.70 | 0.69 | 0.88 | 0.88 |
| Embra6 | 1 | 58 | 114-156 | 122-156 | 0.12 | 0.21 | 0.92 | 0.88 | 0.71 | 0.67 | 0.92 | 0.89 |
| Embra70 | 1 | 56 | 138-170 | 138-170 | 0.20 | 0.19 | 0.87 | 0.87 | 0.88 | 0.63 | 0.88 | 0.88 |
| Embra12 | 1 | 59 | 114-150 | 114-150 | 0.28 | 0.14 | 0.83 | 0.91 | 0.80 | 0.77 | 0.84 | 0.91 |
| Embra35 | 1 | 59 | 202-300 | 202-286 | 0.10 | 0.16 | 0.94 | 0.93 | 0.40 | 0.60 | 0.94 | 0.93 |
| Embra100 | 1 | 61 | 216-276 | 216-260 | 0.14 | 0.13 | 0.92 | 0.92 | 0.84 | 0.83 | 0.93 | 0.93 |
| Eg117 | 1 | 60 | 180-246 | 180-238 | 0.21 | 0.29 | 0.87 | 0.84 | 0.73 | 0.63 | 0.88 | 0.85 |
| En10 | 1 | 60 | 136-180 | 136-180 | 0.24 | 0.19 | 0.80 | 0.89 | 0.38 | 0.35 | 0.83 | 0.90 |
| Embra55 | 2 | 50 | 128-184 | 138-184 | 0.36 | 0.19 | 0.76 | 0.86 | 0.43 | 0.43 | 0.79 | 0.87 |
| Embra63 | 2 | 58 | 176-248 | 176-230 | 0.16 | 0.22 | 0.91 | 0.88 | 0.48 | 0.50 | 0.91 | 0.89 |
| Embra172 | 2 | 59 | 278-318 | 278-318 | 0.25 | 0.21 | 0.83 | 0.83 | 0.43 | 0.56 | 0.85 | 0.85 |
| Embra43 | 2 | 56 | 102-148 | 102-136 | 0.20 | 0.13 | 0.91 | 0.91 | 0.88 | 0.56 | 0.91 | 0.92 |
| Embra207 | 2 | 49 | 208-300 | 212-264 | 0.14 | 0.14 | 0.92 | 0.92 | 0.82 | 0.71 | 0.92 | 0.93 |
| Embra195 | 2 | 48 | 196-270 | 196-262 | 0.11 | 0.10 | 0.92 | 0.93 | 0.87 | 0.67 | 0.93 | 0.93 |
| Embra227 | 3 | 62 | 196-324 | 290-318 | 0.19 | 0.21 | 0.85 | 0.82 | 0.51 | 0.84 | 0.87 | 0.84 |
| Embra122 | 3 | 56 | 110-168 | 110-168 | 0.16 | 0.18 | 0.92 | 0.90 | 0.90 | 0.75 | 0.93 | 0.90 |
| Embra77 | 3 | 58 | 250-340 | 262-330 | 0.19 | 0.17 | 0.89 | 0.88 | 0.60 | 0.47 | 0.90 | 0.89 |
| Eg24 | 3 | 60 | 162-170 | 162-170 | 0.68 | 0.77 | 0.44 | 0.36 | 0.49 | 0.27 | 0.49 | 0.38 |
| Embra19 | 4 | 59 | 138-186 | 138-186 | 0.16 | 0.14 | 0.90 | 0.89 | 0.56 | 0.67 | 0.91 | 0.90 |
| Embra78 | 4 | 59 | 120-166 | 122-156 | 0.22 | 0.40 | 0.89 | 0.80 | 0.68 | 0.69 | 0.90 | 0.81 |
| Embra66 | 4 | 54 | 142-190 | 142-190 | 0.14 | 0.20 | 0.90 | 0.90 | 0.73 | 0.71 | 0.91 | 0.90 |
| Embra36 | 4 | 55 | 232-282 | 242-282 | 0.22 | 0.17 | 0.87 | 0.89 | 0.62 | 0.78 | 0.88 | 0.90 |
| Embra179 | 4 | 56 | 130-156 | 130-156 | 0.26 | 0.24 | 0.82 | 0.87 | 0.59 | 0.69 | 0.84 | 0.88 |
| Embra41 | 5 | 59 | 172-222 | 174-222 | 0.12 | 0.13 | 0.93 | 0.93 | 0.83 | 0.90 | 0.93 | 0.93 |
| Embra143 | 5 | 60 | 106-150 | 106-150 | 0.23 | 0.25 | 0.88 | 0.88 | 0.73 | 0.82 | 0.89 | 0.89 |
| Embra54 | 5 | 58 | 122-160 | 122-158 | 0.18 | 0.30 | 0.88 | 0.85 | 0.54 | 0.67 | 0.89 | 0.86 |
| Embra9 | 5 | 55 | 120-170 | 126-148 | 0.21 | 0.26 | 0.83 | 0.83 | 0.53 | 0.74 | 0.85 | 0.85 |
| Embra24 | 5 | 56 | 142-174 | 142-174 | 0.29 | 0.38 | 0.79 | 0.80 | 0.43 | 0.85 | 0.81 | 0.81 |
| Embra168 | 5 | 55 | 68-94 | 68-88 | 0.36 | 0.46 | 0.68 | 0.58 | 0.81 | 0.80 | 0.72 | 0.65 |
| Embra5 | 5 | 55 | 92-194 | 92-194 | 0.11 | 0.13 | 0.93 | 0.91 | 0.78 | 0.83 | 0.94 | 0.92 |
| Embra28 | 6 | 56 | 130-176 | 130-176 | 0.22 | 0.29 | 0.86 | 0.84 | 0.90 | 0.83 | 0.87 | 0.86 |
| Embra8 | 6 | 55 | 136-180 | 136-180 | 0.11 | 0.19 | 0.93 | 0.90 | 0.93 | 0.73 | 0.93 | 0.91 |
| Embra50 | 6 | 58 | 110-144 | 110-144 | 0.24 | 0.26 | 0.87 | 0.85 | 0.55 | 0.71 | 0.88 | 0.87 |
| Embra25 | 6 | 60 | 246-290 | 250-290 | 0.29 | 0.18 | 0.80 | 0.85 | 0.68 | 0.80 | 0.83 | 0.87 |
| Embra20 | 6 | 59 | 122-162 | 128-162 | 0.17 | 0.22 | 0.90 | 0.87 | 0.85 | 0.76 | 0.91 | 0.88 |
| Embra167 | 7 | 49 | 98-152 | 98-152 | 0.17 | 0.26 | 0.91 | 0.88 | 0.66 | 0.40 | 0.91 | 0.88 |
| Embra7 | 7 | 57 | 144-166 | 144-166 | 0.18 | 0.19 | 0.86 | 0.86 | 0.92 | 0.83 | 0.88 | 0.87 |
| Embra226 | 7 | 64 | 162-216 | 168-216 | 0.14 | 0.21 | 0.90 | 0.89 | 0.76 | 0.89 | 0.91 | 0.90 |
| Embra119 | 8 | 60 | 110-158 | 110-158 | 0.25 | 0.30 | 0.86 | 0.78 | 0.68 | 0.69 | 0.87 | 0.80 |
| Embra3 | 8 | 57 | 120-142 | 122-142 | 0.41 | 0.49 | 0.71 | 0.57 | 0.66 | 0.71 | 0.74 | 0.63 |
| Embra17 | 9 | 57 | 206-266 | 206-266 | 0.21 | 0.19 | 0.87 | 0.90 | 0.71 | 0.75 | 0.88 | 0.91 |
| Embra18 | 9 | 56 | 114-138 | 114-132 | 0.22 | 0.28 | 0.82 | 0.81 | 0.61 | 0.72 | 0.84 | 0.83 |
| Embra204 | 9 | 55 | 134-170 | 140-170 | 0.18 | 0.21 | 0.87 | 0.88 | 0.50 | 0.64 | 0.88 | 0.89 |
| Embra58 | 9 | 60 | 134-194 | 134-194 | 0.11 | 0.12 | 0.93 | 0.93 | 0.93 | 0.92 | 0.93 | 0.93 |
| Embra10 | 10 | 60 | 116-150 | 116-148 | 0.16 | 0.17 | 0.90 | 0.89 | 0.85 | 0.83 | 0.91 | 0.90 |
| Embra23 | 10 | 61 | 108-146 | 108-146 | 0.22 | 0.20 | 0.89 | 0.90 | 0.74 | 0.90 | 0.90 | 0.91 |
| Embra61 | 10 | 58 | 168-194 | 168-194 | 0.15 | 0.17 | 0.89 | 0.89 | 0.43 | 0.43 | 0.90 | 0.90 |
| Embra101 | 10 | 56 | 110-156 | 110-148 | 0.22 | 0.26 | 0.88 | 0.85 | 0.92 | 0.82 | 0.89 | 0.86 |
| Embra40 | 10 | 57 | 110-146 | 110-144 | 0.20 | 0.20 | 0.88 | 0.88 | 0.83 | 0.88 | 0.89 | 0.89 |
| Embra155 | 10 | 62 | 150-194 | 150-190 | 0.28 | 0.32 | 0.87 | 0.84 | 0.72 | 0.64 | 0.88 | 0.85 |
| Embra29 | 11 | 57 | 226-300 | 232-300 | 0.17 | 0.12 | 0.91 | 0.93 | 0.88 | 0.88 | 0.92 | 0.93 |
| Embra39 | 11 | 58 | 124-158 | 124-148 | 0.18 | 0.15 | 0.89 | 0.89 | 0.90 | 0.81 | 0.90 | 0.90 |
| Embra2 | 11 | 57 | 112-150 | 112-150 | 0.44 | 0.33 | 0.71 | 0.77 | 0.59 | 0.70 | 0.74 | 0.80 |
| Eg128 | 11 | 48 | 160-200 | 164-200 | 0.13 | 0.17 | 0.90 | 0.87 | 0.63 | 0.61 | 0.91 | 0.88 |
| Embra243 | - | 56 | 134-204 | 134-204 | 0.55 | 0.28 | 0.60 | 0.79 | 0.46 | 0.69 | 0.64 | 0.81 |
| Embra207 | - | 49 | 220-248 | 220-246 | 0.39 | 0.38 | 0.75 | 0.79 | 0.56 | 0.65 | 0.78 | 0.81 |
| Eg16 | - | 60 | 132-154 | 132-154 | 0.16 | 0.16 | 0.90 | 0.89 | 0.71 | 0.75 | 0.91 | 0.90 |
| Embra72 | - | 59 | 148-170 | 148-170 | 0.54 | 0.34 | 0.57 | 0.77 | 0.51 | 0.40 | 0.63 | 0.79 |
| CD669383 | - | 60 | 370-422 | 370-422 | 0.30 | 0.33 | 0.77 | 0.75 | 0.63 | 0.63 | 0.80 | 0.79 |
| CD668471 | - | 60 | 138-168 | 138-168 | 0.29 | 0.41 | 0.80 | 0.78 | 0.76 | 0.84 | 0.82 | 0.79 |
| CD668519 | - | 60 | 148-168 | 148-168 | 0.53 | 0.78 | 0.63 | 0.37 | 0.95 | 0.44 | 0.67 | 0.38 |
| CD668704 | - | 60 | 108-158 | 108-158 | 0.12 | 0.24 | 0.92 | 0.89 | 0.66 | 0.70 | 0.92 | 0.89 |

LG – ‘Linkage group’; EC – ‘*E.camaldulensis’*; ET – ‘*E.tereticornis’*
