# Supplementary material for: Species Discrimination, Population Structure and Linkage Disequilibrium in Eucalyptus camaldulensis and Eucalyptus tereticornis Using SSR Markers
Source: PLoS One. 2011 Dec 7;6(12):e28252. doi: 10.1371/journal.pone.0028252 (PMC3233572; doi:10.1371/journal.pone.0028252)
Supplement: Table S2 — Details on eucalypts accessions used in this study. (DOC) [file pone.0028252.s002.doc]

**Table S2: Details on eucalypts accessions used in this study**

| **S.No** | **Name of the** | **Subspecies** | **Accession** | **Provenance name** | **Country** | **Trial location** |
| --- | --- | --- | --- | --- | --- | --- |
|  | **Species** |  | **ID** |  | **of origin** |  |
| 1 | *E.camadulensis* | *acuminata* | 31 | Morehead River | Australia | SSO, Satyavedu |
| 2 | *E.camadulensis* | - | 75 | Bulked | Australia | SPA, Pudukottai |
| 3 | *E.camadulensis* | - | 66 | Bulked | Australia | SPA, Pudukottai |
| 4 | *E.camadulensis* | - | 198 | Bulked | Australia | SPA, Panampalli |
| 5 | *E.camadulensis* | - | 196 | Bulked | Australia | SPA, Panampalli |
| 6 | *E.camadulensis* | - | 76 | Bulked | Australia | SPA, Pudukottai |
| 7 | *E.camadulensis* | - | 69 | Bulked | Australia | SPA, Pudukottai |
| 8 | *E.camadulensis* | - | 63 | Bulked | Australia | SPA, Pudukottai |
| 9 | *E.camadulensis* | - | 73 | Bulked | Australia | SPA, Pudukottai |
| 10 | *E.camadulensis* | - | 70 | Bulked | Australia | SPA, Pudukottai |
| 11 | *E.camadulensis* | *simulata* | 123 | Kennedy river | Australia | PRS, Pudukottai |
| 12 | *E.camadulensis* | *simulata* | 100 | Kennedy river | Australia | PRS, Pudukottai |
| 13 | *E.camadulensis* | *simulata* | 111 | Kennedy river | Australia | PRS, Pudukottai |
| 14 | *E.camadulensis* | *simulata* | 124 | Kennedy river | Australia | PRS, Pudukottai |
| 15 | *E.camadulensis* | *simulata* | 118 | Kennedy river | Australia | PRS, Pudukottai |
| 16 | *E.camadulensis* | *simulata* | 116 | Kennedy river | Australia | PRS, Pudukottai |
| 17 | *E.camadulensis* | *simulata* | 115 | Kennedy river | Australia | PRS, Pudukottai |
| 18 | *E.camadulensis* | *simulata* | 101 | Kennedy river | Australia | PRS, Pudukottai |
| 19 | *E.camadulensis* | *simulata* | 113 | Kennedy river | Australia | PRS, Pudukottai |
| 20 | *E.camadulensis* | *simulata* | 172 | Kennedy River | Australia | PRS, Pudukottai |
| 21 | *E.camadulensis* | *simulata* | 53 | Kennedy river | Australia | PRS, Pudukottai |
| 22 | *E.camadulensis* | *simulata* | 186 | Kennedy River | Australia | SSO, Panampalli |
| 23 | *E.camadulensis* | *simulata* | 131 | Laura River | Australia | PRS, Pudukottai |
| 24 | *E.camadulensis* | *simulata* | 217 | Palmer River | Australia | SPA, Pudukottai |
| 25 | *E.camadulensis* | *acuminata* | 187 | Gilbert River | Australia | SSO, Panampalli |
| 26 | *E.camadulensis* | *acuminata* | 16 | Gilbert River | Australia | SSO, Satyavedu |
| 27 | *E.camadulensis* | - | 154 | Wrotham | Australia | SSO, Pudukottai |
| 28 | *E.camadulensis* | *acuminata* | 26 | Petford | Australia | SSO, Satyavedu |
| 29 | *E.camadulensis* | *obtusa* | 15 | Katherine | Australia | SSO, Satyavedu |
| 30 | *E.camadulensis* | *obtusa* | 191 | Katherine | Australia | SSO, Panampalli |
| 31 | *E.camadulensis* | *obtusa* | 19 | Katherine | Australia | SSO, Satyavedu |
| 32 | *E.camadulensis* | *obtusa* | 17 | Katherine | Australia | SSO, Satyavedu |
| 33 | *E.camadulensis* | *obtusa* | 1 | Katherine | Australia | SSO, Satyavedu |
| 34 | *E.camadulensis* | *obtusa* | 9 | Katherine | Australia | SSO, Satyavedu |
| 35 | *E.camadulensis* | *obtusa* | 136 | Katherine | Australia | SSO, Pudukottai |
| 36 | *E.camadulensis* | *obtusa* | 23 | Victoria River | Australia | SSO, Satyavedu |
| 37 | *E.camadulensis* | *obtusa* | 188 | Victoria River | Australia | SSO, Panampalli |
| 38 | *E.camadulensis* | *obtusa* | 14 | Victoria River | Australia | SSO, Satyavedu |
| 39 | *E.camadulensis* | *obtusa* | 10 | Victoria River | Australia | SSO, Satyavedu |
| 40 | *E.camadulensis* | *obtusa* | 7 | Victoria River | Australia | SSO, Satyavedu |
| 41 | *E.tereticornis* | *tereticornis* | 23-12-18 | Orobay | PNG | SSO, Karunya |
| 42 | *E.tereticornis* | *tereticornis* | 25-15-20 | Orobay | PNG | SSO, Karunya |
| 43 | *E.tereticornis* | *tereticornis* | 30 -19-17 | Orobay | PNG | SSO, Karunya |
| 44 | *E.tereticornis* | *tereticornis* | 35-25-18 | Orobay | PNG | SSO, Karunya |
| 45 | *E.tereticornis* | *tereticornis* | 18-8-18 | Orobay | PNG | SSO, Karunya |
| 46 | *E.tereticornis* | *tereticornis* | 18-6-18 | Orobay | PNG | SSO, Karunya |
| 47 | *E.tereticornis* | *tereticornis* | 3-26-20 | Orobay | PNG | SSO, Karunya |
| 48 | *E.tereticornis* | *tereticornis* | 3-25-20 | Orobay | PNG | SSO, Karunya |
| 49 | *E.tereticornis* | *tereticornis* | 8-5-14 | Kupiano | PNG | SSO, Karunya |
| 50 | *E.tereticornis* | *tereticornis* | 14-15-15 | Kupiano | PNG | SSO, Karunya |
| 51 | *E.tereticornis* | *tereticornis* | 34-18-14 | Kupiano | PNG | SSO, Karunya |
| 52 | *E.tereticornis* | *tereticornis* | 35-18-12 | Kupiano | PNG | SSO, Karunya |
| 53 | *E.tereticornis* | *tereticornis* | 26-3-14 | Kupiano | PNG | SSO, Karunya |
| 54 | *E.tereticornis* | *tereticornis* | 32-12-23 | Sogeri plateau | PNG | SSO, Karunya |
| 55 | *E.tereticornis* | *tereticornis* | 34-2-49 | North Kennedy River | Australia | SSO, Karunya |
| 56 | *E.tereticornis* | *tereticornis* | 28-12-50 | North Kennedy River | Australia | SSO, Karunya |
| 57 | *E.tereticornis* | *tereticornis* | 36-22-51 | North Kennedy River | Australia | SSO, Karunya |
| 58 | *E.tereticornis* | *tereticornis* | 7-13-40 | Ravenshoe | Australia | SSO, Karunya |
| 59 | *E.tereticornis* | *tereticornis* | 7-14-40 | Ravenshoe | Australia | SSO, Karunya |
| 60 | *E.tereticornis* | *tereticornis* | 7-15-40 | Ravenshoe | Australia | SSO, Karunya |
| 61 | *E.tereticornis* | *tereticornis* | 11-4-41 | S Cardwell | Australia | SSO, Karunya |
| 62 | *E.tereticornis* | *tereticornis* | 15-4-41 | S Cardwell | Australia | SSO, Karunya |
| 63 | *E.tereticornis* | *tereticornis* | 32-10-41 | S Cardwell | Australia | SSO, Karunya |
| 64 | *E.tereticornis* | *tereticornis* | 10-17-8 | Cardwell | Australia | SSO, Karunya |
| 65 | *E.tereticornis* | *tereticornis* | 8-16-6 | SW of Mt.Garnet | Australia | SSO, Karunya |
| 66 | *E.tereticornis* | *tereticornis* | 16-21-6 | SW of Mt.Garnet | Australia | SSO, Karunya |
| 67 | *E.tereticornis* | *tereticornis* | 31-25-45 | SW of Mt.Garnet | Australia | SSO, Karunya |
| 68 | *E.tereticornis* | *tereticornis* | 34-24-44 | SW of Mt.Garnet | Australia | SSO, Karunya |
| 69 | *E.tereticornis* | *tereticornis* | 25-9-4 | S Helenvale | Australia | SSO, Karunya |
| 70 | *E.tereticornis* | *tereticornis* | 36-3-5 | S Helenvale | Australia | SSO, Karunya |
| 71 | *E.tereticornis* | *tereticornis* | 32-16-30 | 5-15km S Helenvale | Australia | SSO, Karunya |
| 72 | *E.tereticornis* | *tereticornis* | 3-13-35 | 5-15km S Helenvale | Australia | SSO, Karunya |
| 73 | *E.tereticornis* | *tereticornis* | 7-12-30 | 5-15km S Helenvale | Australia | SSO, Karunya |
| 74 | *E.tereticornis* | *tereticornis* | 15-20-1 | SW MT Garnet | Australia | SSO, Karunya |
| 75 | *E.tereticornis* | *tereticornis* | 21-11-1 | SW MT Garnet | Australia | SSO, Karunya |
| 76 | *E.tereticornis* | *Landrace* | CC - 4 | - | India | SSO, Karunya |
| 77 | *E.tereticornis* | *Landrace* | CC - 3 | - | India | SSO, Karunya |
| 78 | *E.tereticornis* | *Landrace* | CC - 7 | - | India | SSO, Karunya |
| 79 | *E.tereticornis* | *Landrace* | CC - 10 | - | India | SSO, Karunya |
| 80 | *E.tereticornis* | *Landrace* | SMD-7 | Sethumadai | India | SSO, Karunya |
| 81 | *E.tereticornis* | *Landrace* | SMD 7 | Sethumadai | India | SSO, Karunya |
| 82 | *E.tereticornis* | *Landrace* | ET 9 - 3 | Mettupalayam | India | SSO, Karunya |
| 83 | *E.tereticornis* | *Landrace* | 231 | Mettupalayam | India | SSO, Karunya |
| 84 | F1 hybrid | F1 individual | Et x Eg | - | India | Panampalli |
| 85 | F1 hybrid | F1 individual | Et x Eg | - | India | Panampalli |
| 86 | F1 hybrid | F1 individual | Et x Eg | - | India | Panampalli |
| 87 | F1 hybrid | F1 individual | Et x Eu | - | India | Panampalli |
| 88 | F1 hybrid | F1 individual | Et x Ep | - | India | Panampalli |
| 89 | F1 hybrid | F1 individual | Et x Ep | - | India | Panampalli |
| 90 | F1 hybrid | F1 individual | Et x Eu | - | India | Panampalli |
| 91 | F1 hybrid | F1 individual | Et x Eu | - | India | Panampalli |
| 92 | F1 hybrid | F1 individual | Et x Eu | - | India | Panampalli |
| 93 | F1 hybrid | F1 individual | Et x Eu | - | India | Panampalli |

SSO- ‘Seedling seed orchard’; SPA –‘Seed production area’; PRS- ‘Provenance resource stand’ PNG – ‘Papua New Guinea’; Et – ‘*E. tereticornis’*; Eg – ‘*E. grandis’*; Eu – ‘*E. urophylla’*; Ep – ‘*E. pellita’*
